# Defect Dipole Behaviors on the Strain Performances of Bismuth Sodium Titanate-Based Lead-Free Piezoceramics

**DOI:** 10.3390/ma16114008

**Published:** 2023-05-26

**Authors:** Yiyi Wang, Pu Wang, Laijun Liu, Yuyin Wang, Yingying Zhao, Wenchao Tian, Xiao Liu, Fangyuan Zhu, Jing Shi

**Affiliations:** 1 School of Materials Science and Engineering, Xi’an University of Science and Technology, Xi’an 710054, China; 2 Key Laboratory of New Processing Technology for Nonferrous Metal & Materials (MOE), College of Materials Science and Engineering, Guilin University of Technology, Guilin 541006, China; ljliu2@163.com; 3 Key Laboratory of Electronic Equipment Structure Design (MOE), School of Mechano-Electronic Engineering, Xidian University, Xi’an 710071, China; wctian@xidian.edu.cn; 4 Shanghai Synchrotron Radiation Facility, Shanghai Advanced Research Institute, Chinese Academy of Sciences, Shanghai 201204, China; zhufy@sari.ac.cn

**Keywords:** bismuth sodium titanate, strain, defect dipole, polarization, hysteresis, asymmetric, ceramics

## Abstract

Bismuth sodium titanate (BNT)-based, lead-free piezoelectric materials have been extensively studied due to their excellent strain characteristics and environmental friendliness. In BNTs, the large strain (*S*) usually requires a relatively large electric field (*E*) excitation, resulting in a low inverse piezoelectric coefficient d_33_* (*S*/*E*). Moreover, the hysteresis and fatigue of strain in these materials have also been bottlenecks impeding the applications. The current common regulation method is chemical modification, which mainly focuses on forming a solid solution near the morphotropic phase boundary (MPB) by adjusting the phase transition temperature of the materials, such as BNT-BaTiO_3_, BNT-Bi_0.5_K_0.5_TiO_3_, etc., to obtain a large strain. Additionally, the strain regulation based on the defects introduced by the acceptor, donor, or equivalent dopant or the nonstoichiometry has proven effective, but its underlying mechanism is still ambiguous. In this paper, we review the generation of strain and then discuss it from the domain, volume, and boundary effect perspectives to understand the defect dipole behavior. The asymmetric effect caused by the coupling between defect dipole polarization and ferroelectric spontaneous polarization is expounded. Moreover, the defect effect on the conductive and fatigue properties of BNT-based solid solutions is described, which will affect the strain characteristics. The optimization approach is appropriately evaluated while there are still challenges in the full understanding of the defect dipoles and their strain output, in which further efforts are needed to achieve new breakthroughs in atomic-level insight.

## 1. Introduction

Piezoelectric material is an important functional material that constitutes the core component of electronic devices widely employed in new technology fields, including actuators, sensors, and transducers, since it can realize the conversion of electrical energy and mechanical energy. Lead-based piezoelectric materials, represented by Pb(Zr_1−*x*_Ti*_x_*)O_3_ (PZT), have long occupied the commercial market because of their excellent strain performance [1,2]. Nevertheless, owing to the great harm lead does to health and the ecological environment [3], many countries have begun to legislate to limit the content of lead in electronic products. As a result, there is an urgent demand for research on lead-free piezoelectric materials to satisfy market needs and environmental requirements [4]. A variety of lead-free piezoelectric materials (e.g., Bi_0.5_Na_0.5_TiO_3_ (BNT), K_0.5_Na_0.5_NbO_3_ (KNN), and BaTiO_3_ (BT)) have been found to possess electric-induced strain behavior [2,5], and various methods have been developed to achieve improved strain properties (Table 1). The electric field-induced strain in perovskite materials includes intrinsic and extrinsic contributions. Intrinsic contributions originate from electrostriction and piezoelectric effects, while extrinsic contributions are produced by domain switching and phase transformation. Among lead-free piezoelectric materials, BNT is considered one of the promising alternatives to replace lead-based composite ceramics in the application of actuators because the giant strain (*S* > 0.45%) can be regulated comparable to lead-based materials near its morphotropic phase boundary (MPB) [6]. Monitoring the volume changes in strains in BNT indicates that the origin of the large strain is due to the relaxor-ferroelectric transition [7]. There are more strain mechanisms in BNT-based materials. A giant electric field-induced strain of 0.87% is reproducibly observed for BNT-BKT-BT crystals, originating from 90° domain switching [8]. The electrostrictive effect makes the main contribution to the hysteresis-free strain [9]. BNTs with negligible hysteresis in strain always have a small d_33_*. The contradiction is raised by balancing the large strain and low hysteresis, as shown in Table 1. Interestingly, the abnormal giant d_33_* in the high strain side of the asymmetric bipolar strain curves has recently been reported in several works where the defect dipole plays a critical role associated with either relatively large or moderate hysteresis.

BNT exhibits a complex perovskite structure in which Bi^3+^ and Na^+^ coexist in the A-site at the vertex of the unit cell [35]. Its disordered distribution forms a random field with continuous symmetric order parameters, which will destroy the transition from the system to the long-range order state. When the temperature is below the Burns temperature, the formation of polar nano-regions (PNRs) in the system is attributed to the inconsistency of the internal structure and charge of the system caused by the random field in the BNT, which induces the material into the ergodic relaxor phase (ER). The PNRs are polarized into ferroelectric macrodomains if a sufficiently large electric field is applied, and the material will return to the initial phase structure after the electric field is removed, which favors the significant reversible strain due to the phase transition between the ferroelectric phase (FE) and ER [36]. Although the relaxor-ferroelectric crossover is the critical key for the giant electro-strain, the strain mechanism can also be investigated from a micro perspective. Meanwhile, the change in ion displacement of A/B sites and oxygen octahedral tilt (anti-phase tilt *a*^−^*a*^−^*a*^−^ and in-phase tilt *a*^0^*a*^0^*c*^+^) are accompanied, and the average structure and local structure are changed accordingly. When the temperature continues to decrease below the relaxation-ferroelectric phase transition temperature (*T*_F–R_), the material transforms into the nonergodic relaxation phase (NR). At this time, the nano-domain appears in the system, and the electric field is large enough to cause the phase transition of the BNT-based material from the NR to the FE, also accompanied by the structure change above [37]. However, when the electric field is removed, the material structure remains in the ferroelectric phase. This irreversible characteristic of the NR to FE phase leads to the one-time large strain of the material. Through shifting the high-temperature ER phase to room temperature by composition regulation, a large reversible strain at room temperature can be obtained by utilizing the electro-induced ER-EF transformation.

Generally, the large strain of BNT-based materials is triggered by a large electric field [6], which makes its *S*/*E* smaller than that of KNN and BT-based materials (>1000 pm/V), while the maximum unipolar strains of these two materials rarely exceed 0.4% [38,39]. The *S*/*E* of BNT-based materials needs to be further ameliorated, which should be considered under the condition of a reduced applied electric field. On the other hand, the large hysteresis Δ*S*/*S*_max_ > 50% (Δ*S* is the difference in the strain at the electric field *E* = *E*_max_/2 during the withdrawal and application of the electric field) [40] and inferior stability [41,42] are the main obstacles in the *S-E* curves. Furthermore, the fatigue properties of BNT-based materials should also be optimized in an effort to ensure the reliability of the application [42].

Currently, the common strategy to synthesize materials with excellent strain properties, including ferroelectric phase/relaxation phase composites, that is, ferroelectric phase/relaxation phase BNT-based materials, is to form 0–3 type composites to reduce the strain hysteresis of BNT [43] or polar semiconductor/ferroelectric materials to increase the depolarization temperature *T*_d_ [44]. For instance, Lee et al. disclose that the coupling of the polarization with the nonpolar to ferroelectric phase transition first in Bi_0.5_(Na_0.75_K_0.25_)_0.5_TiO_3_-BiAlO_3_ ferroelectric/relaxor ceramics can enhance the strain properties [45]. Or by adjusting the process parameters, a core-shell structure is constructed, and the core and shell have different formation temperatures, resulting in metastable heterogeneous microstructures that lead to a change in strain [46]. Moreover, by adopting grain orientation technology, the direction of grain growth can be shifted, and the performance of ceramics will be improved [47]. The introduction of defects, including vacancies, lattice interstitial ions, and ions that replace normal lattices through equivalent or heterovalent substitution at A/B sites, can generate defect dipoles with other ions to pin the domain wall and hinder the switching of ferroelectric domains under the external field, thereby allowing the structure of the material to be adjusted to achieve performance optimization.

Most of the modified systems focus on the formation of solid solutions at MPB to regulate the temperature of phase transition between the ER and the FE of the material to obtain large strain [48], and only a few studies have explored the strain regulation mechanism from defects. Thus, it is necessary to clarify the internal mechanism according to the origin of the strain. Therefore, this review elaborates on the regulation mechanism of defect dipoles on strain properties, and some unusual phenomena of *S-E* loops induced by the coupling of defect dipoles and spontaneous polarization are demonstrated. The strain properties of BNT-based solid solutions under the action of defect dipoles are compiled. We summarize the recent research and the primary issues with defect dipoles, and future work is prospected.

## 2. Structural Origin of Strain in BNT-Based Materials

The interaction between the polarization intensity and consequent strain output displays a fascinating research hotspot when applying an electric field to ferroelectric materials. This strain is primarily due to the intrinsic contributions from the inverse piezoelectric effect and electrostriction, which are brought about by the ion displacement from its equilibrium position, resulting in negligible hysteresis of *S-E* curves, which is advantageous for practical applications [49]. The extrinsic contribution includes non-180° domain wall motion and the phase transition produced by electric field-induced paraelectric-ferroelectric phase transition, ferroelectric phase transition, and antiferroelectric-ferroelectric phase transition [49,50]. These will lead to hysteresis and nonlinear *S-E* loops, giving rise to more complicated strain [51].

**Figure 1:**
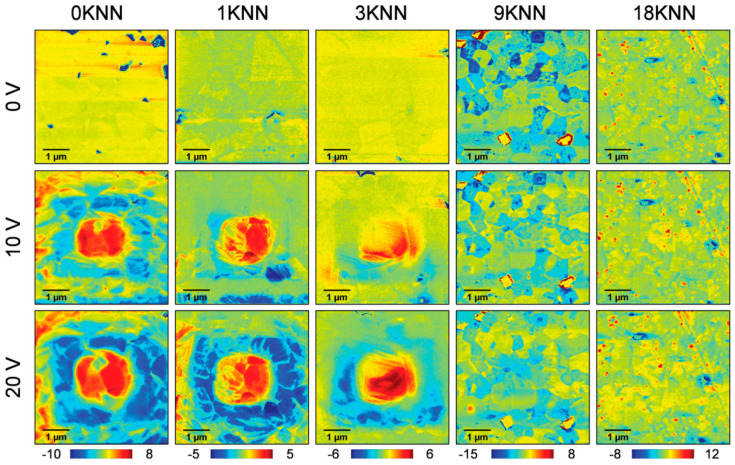
The polarization behavior of 0.94BNT-0.06BT-*x*KNN ceramics observed by PFM. Reproduced with permission from [54].

The preliminary study of the typical ferroelectric material BNT focuses on the large strain of approximately 0.45% measured in 0.91Bi_0.5_Na_0.5_TiO_3_-0.06BaTiO_3_-0.03(K_0.5_Na_0.5_)NbO_3_ (0.91BNT-0.06BT-0.03KNN) ceramics under an electric field of 80 kV/cm. The strain was considered to stem from the antiferroelectric-ferroelectric phase transition because the material showed double hysteresis loop characteristics [6,52,53]. By synchronously measuring the longitudinal and transverse electric strain, as proposed by Jo et al., the large strain in BNT-based materials was considered to attribute the phase transition from the non-polarized phase to the ferroelectric phase [7]. In Figure 1, the in-situ PFM carried out for 0.94BNT-0.06BT-*x*KNN ceramics shows that the repeatable large strain nature is a recoverable feature of the transition from non-polarized to ferroelectric phase under the application of electric fields [54].

**Figure 2:**
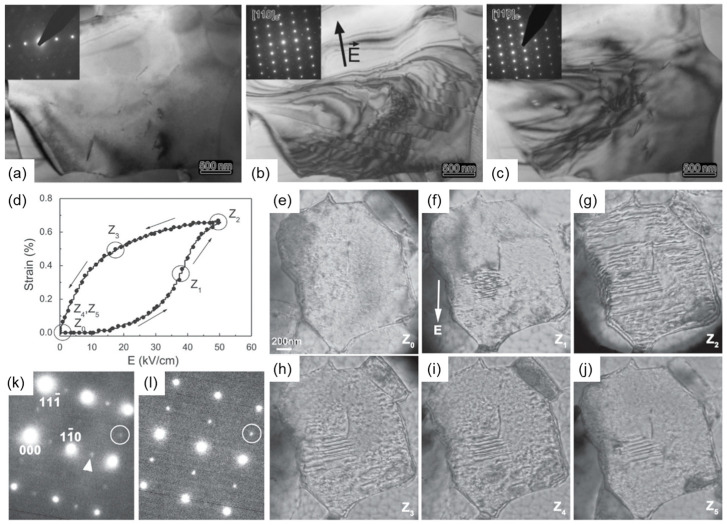
In-situ TEM observation of 0.91BNT-0.06BT-0.03KNN ceramics. (**a**) No electric field is applied. (**b**) Under an electric field of 50 kV/cm. (**c**) Remove the electric field. (**d**) The strain in the BNT-2.5Nb under unipolar fields. The Z_0_–Z_5_ on the curve correspond to the electric field applied by different domain states in (**e**–**j**). The ½{*ooo*} and ½{*ooe*} superlattice diffraction spots are highlighted in (**k**) and (**l**). Reproduced with permission from [10,55].

Kling et al. studied the non-polarized phase utilized in in-situ TEM of 0.91BNT-0.06BT-0.03KNN ceramics. As displayed in Figure 2a–c, the appearance and disappearance of ferroelectric domains by controlling the electric field indicate the phase transition from the non-polarized phase to the ferroelectric phase. The existence of two superlattice reflections in the illustration proves the coexistence of rhombohedral and tetragonal phases in the local structure [55]. In-situ TEM was also carried out by Tan et al. to reveal the evolution process of the domain structure of Bi_0.5_(Na_0.84_K_0.16_)_1/2_)_0.96_Sr_0.04_)(Ti_0.975_Nb_0.025_)O_3_ (BNT-2.5Nb) ceramics, and they obtained the same conclusion, as illustrated in Figure 2d–l [10]. Daniels et al. demonstrated that the average structure induced by the electric field changed from a pseudocubic to a tetragonal phase [56]. Simons found the transition from the pseudocubic phase to mixed ferroelectric rhombohedral and tetragonal phases in BNT-BT by in-situ neutron diffraction [57].

**Figure 3:**
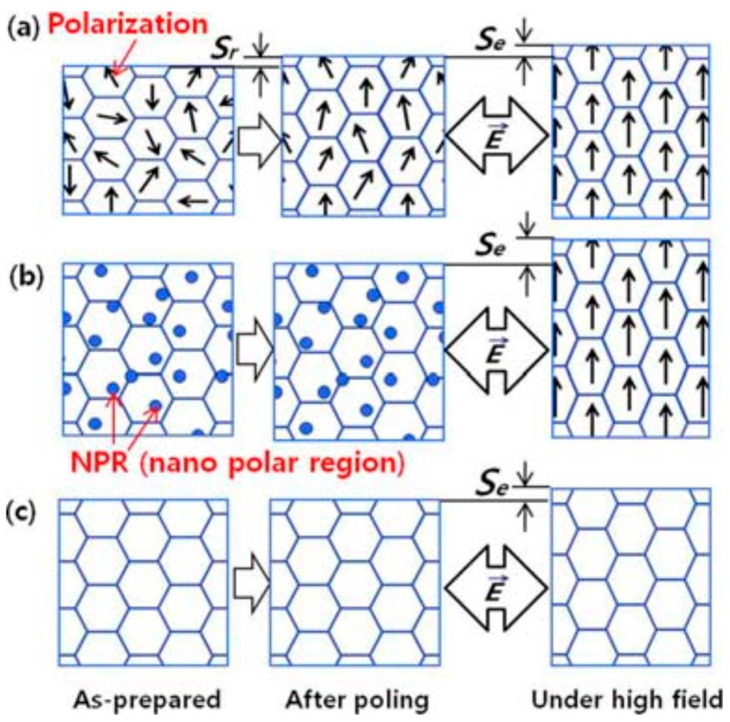
Model of electric field-induced strain in BNT-based materials. (**a**) The ferroelectric specimens will produce *S*r after polarization, and further application of electric field will induce *S*e. (**b**) The specimen with the ferroelectric-nonpolar phase exhibits PNRs, which can produce a giant strain *S*e. (**c**) The nonpolar samples show only electrostrictive under electric fields. Reproduced with permission from [58].

A schematic illustration of electric field-induced nonpolar pseudocubic to polar anisotropic symmetry has been advanced by Lee et al. [58]. As exhibited in Figure 3a, ferroelectric specimens consist of randomly oriented ferroelectric domains that are reoriented after poling, resulting in a remnant strain *S*_r_. Furthermore, an electric field is applied to poled specimens, inducing a piezoelectric strain *S*_e_. In contrast, the specimen with the ferroelectric-nonpolar phase boundary exhibits PNRs in the nonpolar matrix in Figure 3b [58,59]. The PNRs can reversibly transform to the long-range-ordered state under cyclic fields, giving a giant recoverable strain response [60]. The nonpolar samples in Figure 3c show only electrostrictive behavior under electric fields. The specimens have no ferroelectric domain and have a small piezoelectric response. The common feature of these phase transitions is that after the electric field is revoked, both the average structure and the local structure return to their original states. Meanwhile, the microscopic morphology varies with the domain structure at different scales. At this time, the strain caused by the strong field is reversible, mainly arising from the electric field-induced transition from the ER phase to the FE phase.

## 3. Defect Dipole Behavior-Structure-Strain Regulation

**Figure 4:**
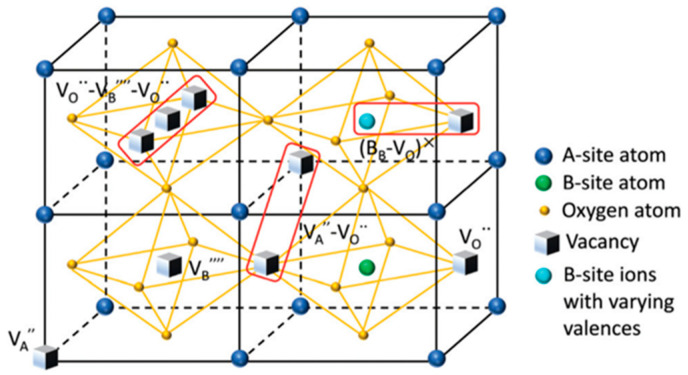
Schematic diagram of defects formed in the perovskite structure. Reproduced with permission from [61].

In perovskite structures, the vacancy will be frequently created during the synthesis and substitution processes and has an important effect on the “hardening”, “softening”, aging, fatigue, and ionic conductivity of the materials [61,62,63]. For instance, the A-site elements of BNT-based materials are easily volatile, and defects will occur in the preparation. In the A/B heterovalent substitution modification, in order to maintain the electricity balance, an A site or oxygen vacancy will also appear, and these charged defects will compound to form defect dipoles under certain conditions [61,64]. As shown in Figure 4, the defect dipoles can act on the domain and phase structure and play a prominent role in the properties of piezoelectric materials.

**Figure 5:**
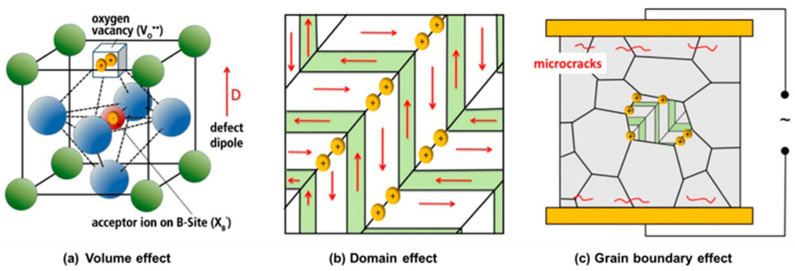
(**a**) Volume effect; (**b**) Domain effect; (**c**) Grain boundary effect of defect dipoles. Reproduced with permission from [66].

The effect of defect dipoles can be divided into three levels: volume effect, domain effect, and grain boundary effect, as exhibited in Figure 5 [65]. The volume effect is associated with the dipoles formed by acceptors and vacancies, which have an electric or elastic dipole moment. The domain effect describes the diffusion of charged defects and the pinning effect of domain walls. The grain boundary effect is related to the interface diffusion of carriers and the formation of a space charge layer. The influence mechanism of the defect dipole is different due to the special temperature field and electric field phase transition of BNT. At present, it is mainly studied in the abovementioned aspects.

### 3.1. Domain and Grain Boundary Effects of Defect Dipoles

Domain wall pinning and morphological changes may occur under the action of the defect dipole [67]. The stability of the ferroelectric phase would be degraded due to the absence of Na^+^ [68] or excessive Bi^3+^ [69] in nonstoichiometric BNT. $V_{Na}^{\prime }$-$V_{O}^{••}$-$V_{Na}^{\prime }$ defect dipoles develop inside the material when Na is absent. This could pin the grain boundaries and result in smaller grains. Meanwhile, the high-mobility $V_{O}^{••}$ is consumed, contributing to the high mobility of domain boundaries. In the Bi-deficient state, the complex defect dipole of 2$(V_{Bi}^{\prime \prime \prime }-V_{O}^{••}{)}^{\prime }$-$V_{O}^{••}$ is hard to form, and $V_{O}^{••}$ becomes movable, promoting the transport of materials to form larger grains during sintering. It is easy to pin the domain boundary and increase the stability of the domain.

**Figure 6:**
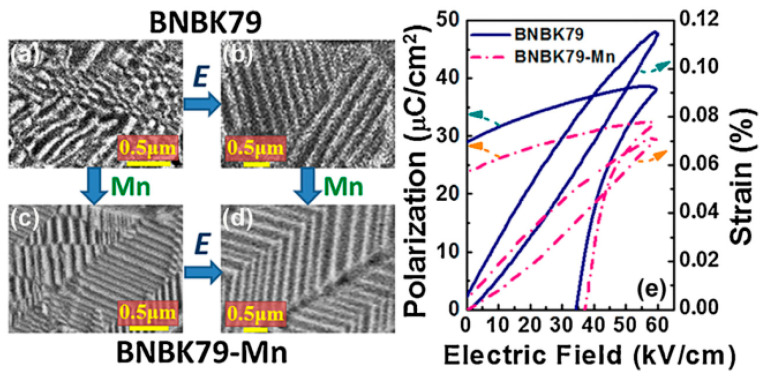
The domain structure of BNBK79 ceramics (**a**,**b**) before Mn doping, (**c**,**d**) after Mn doping, (**a,c**) before polarization, and (**b,d**) after polarization. (**e**) *P-E* and *S-E* curves of two samples. Reproduced with permission from [70].

Qin et al. reported Mn-doped 0.79(Na_0.5_Bi_0.5_)TiO_3_-0.14(K_0.5_Na_0.5_)TiO_3_-0.07BaTiO_3_ (BNBK79) ceramics [70]. The introduction of Mn increases the grain size and changes the domain structure, showing a herringbone complex domain structure, as seen in Figure 6a–d. Meanwhile, the formation of the defect dipole $Mn_{Ti}^{\prime \prime }-V_{O}^{••}$ causes the pinning effect, which hinders domain switching and inflicts the unique dynamic behavior of the *S-E* and *P-E* curves in Figure 6e. Furthermore, Mn was added to the Bi_0.5_Na_0.5_TiO_3_–(Sr_0.7_Bi_0.2□0.1_)TiO_3_ (BNT-SBT) system by Xu et al. to form the $Mn_{Ti}^{\prime \prime }-V_{O}^{••}$ dipole, which can fix the oxygen vacancies in SBT with intrinsic vacancies and weaken the pinning effect of the domain wall, resulting in a larger strain [71]. In the Bi-nonstoichiometric doped BNT-BT, it was found by Qiao et al. that oxygen vacancies pin the ferroelectric domains, reduce the contribution of domain motion, and lead to decreased polarization and strain [72]. Identically, Sasipohn et al. studied Li-doped Bi_0.5_Na_0.5_TiO_3_- BaTiO_3_ (BNT-BT) ceramics. The reduction in *S-E* curves may be caused by the pinning of domain walls by defect dipoles originating from acceptor doping [73]. Therefore, the formation of stable defect dipoles and the consumption of high-mobility $V_{O}^{••}$ are beneficial for the ferroelectric domain activity and will increase the electrical strain of the material. In addition, there is no macroscopic ferroelectric domain but only a nanodomain in the NR phase of BNT-based materials before the electric field is applied. The mechanism of defect dipoles on nanodomains remains to be studied.

### 3.2. Volume Effect of Defect Dipole

**Figure 7:**
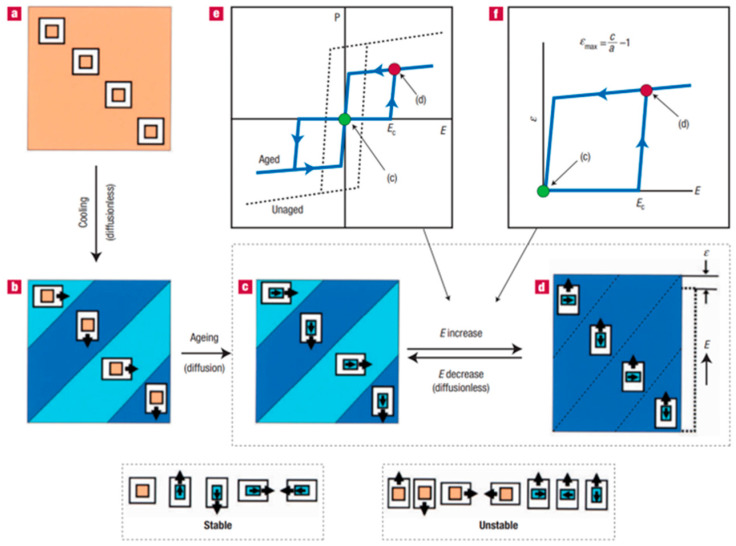
(**a**) Equilibrium cubic paraelectric crystal. (**b**) Multi-domain tetragonal ferroelectric crystal immediately after the cubic-to-tetragonal transition. (**c**) Stable state after aging in the ferroelectric state. (**d**) Unstable state after domain switching from (**c**) by the electric field. (**e**) Double hysteresis loop during reversible domain switching between (**c**) and (**d**). (**f**) Huge recoverable strain during reversible domain switching between (**c**) and (**d**). Reproduced with permission from [75].

The symmetry-consistent short-range order theory (volume effect) of defect dipoles is widely used in other perovskite ferroelectric materials [74,75], i.e., the polarization dipoles will be affected by the symmetry structure of the material itself during the aging process [76,77]. As described in Figure 7, defect dipole polarization (*P_d_*) is generated during aging and aligns along the same direction as spontaneous polarization (*P_s_*). The external electric field can cause the *P_s_* to reorient, while the *P_d_* still keeps its position during such a process. Therefore, when the electric field is removed, the directional alignment of defect dipoles will serve as an internal field to generate the domain recovery force, resulting in a tight “waist” ferroelectric hysteresis loop and increased electric strain [78]. PNRs are distributed in the non-polarized parent phase with little structural distortion, according to the structural features of BNT-based materials in the NR and ER phase regions. The directional alignment behavior of defect dipoles differs from that of macrodomain ferroelectric materials.

#### 3.2.1. Volume Effect in the NR Phase

When Fe ions were added to BNT, Aksel et al. found the formed defect dipole ${\left(Fe_{Ti}^{\prime }-V_{O}^{••}\right)}^{•}$ only played a “medium hardening” role in BNT. They also concluded that it is challenging to supply the driving force for the directional alignment of dipoles because of the approximately cubic structure of BNT-based materials [79,80].

#### 3.2.2. Volume Effect in the Non-Polarized FE Phase Region

A Bi_0.5_Na_0.5_TiO_3_-Bi_0.5_K_0.5_TiO_3_-BaTiO_3_ (BNT-BKT-BT) single crystal in the tetragonal FE phase was thoroughly studied by Teranishi et al. They discovered the tight “waist” hysteresis loops and the significantly large strain (*S* = 0.87%) caused by the dipole composed of A-site and oxygen vacancies [8]. According to Sapper et al., after aging, the hysteresis loop exhibits clamping characteristics associated with the reduction in strain in Fe-substituted ferroelectric tetragonal BNT-BT. The directed alignment of polarized dipoles to stabilize specific ferroelectric phases is assumed [81].

#### 3.2.3. Volume Effect of Polarized BNT-Based Materials

**Figure 8:**
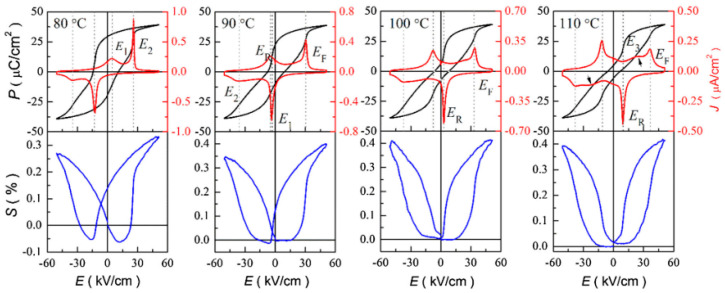
*P-E* and *S-E* curves of Li in Ce-BNKT ceramics. Reproduced with permission from [85].

After aging under polarization, the defect dipoles in the material tend to align in the polarization direction, generating internal tension in that direction that causes the shift of the hysteresis loop and strain. In Mn-substituted lead-based materials, Du and Yan found a large strain memory effect, meaning that the difference in strain does not equal zero when the electric field is absent [82,83]. Since it can keep a specific strain value even in the absence of a constant external electric field, the strain memory effect can be applied to strain memory piezoelectric actuators [84]. The polarized Fe-substituted BNT-1BT material is in the rhombohedral phase as a result of the NR-FE phase transition brought on by polarization, and the defect dipole can shift the hysteresis loop [81]. Shi et al. reported a defect dipole induced by partial Li^+^ entering the B site in Li-substituted (Bi_0.5_Na_0.4_K_0.1_)_0.98_Ce_0.02_TiO_3_ (Li, Ce-BNKT) in which the ${\left(Li_{Ti}^{\prime \prime \prime }-V_{O}^{••}\right)}^{\prime }$ will create an asymmetric shift of the hysteresis loop and the strain curve in the polarized FE phase region that causes a large strain memory effect. When the temperature is higher than the depolarization temperature within a certain range, although it has entered the ER phase, the hysteresis loop and strain still retain asymmetry, as displayed in Figure 8, as a result of the directional pinning action of defect dipoles in the remaining ferroelectric phase [85].

**Figure 9:**
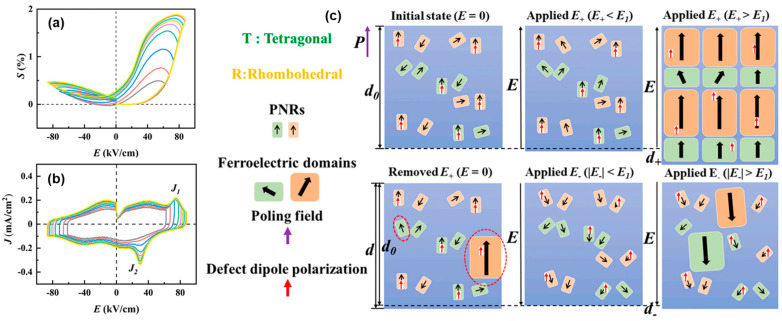
Bipolar (**a**) *S-E* and (**b**) *J-E* loops of the poled BNTBT-2SS sample. (**c**) The domain transformation process after polarization under different electric fields. Reproduced with permission from [16].

Jia et al. obtained a giant asymmetric strain in (Bi_0.5_Na_0.5_)_0.94_Ba_0.06_Ti_0.98_(Sn_0.5_Sb_0.4□0.1_)_0.02_O_3_ (BNTBT-2SS) solid solution by manipulating the defect dipole [16]. The change of current peak and strain under the action of the electric field indicates the transition between PNRs and ferroelectric domains, as shown in Figure 9a,b. When an electric field along the polarization direction (*E_+_*) is applied, *P_d_* switches to being aligned with the polarization field after polarization. It is beneficial for the polarization rotation of the nearby PNRs [86], which promotes the formation of ferroelectric domains parallel to or close to the *P_d_* direction, resulting in a larger strain. Correspondingly, the transition of PNRs to ferroelectric domains is hindered by *P_d_* when an electric field is applied in the opposite direction of polarization, resulting in a small strain.

#### 3.2.4. Volume Effect in the ER Phase

Cao et al. discovered the pinched phenomenon of the hysteresis loop caused by the orientation arrangement of the defect dipoles in the Mn-substituted BNT-BT-ST ceramics and obtained a very small hysteresis loop and strain. Additionally, the composition of BNT-BT-ST is in the ER phase, which is much higher than the depolarization temperature [87]. They continue to achieve enhanced strain in a tetragonal 0.7BNT-0.3ST (BNST) doped with Mn under a low electric field, which is caused by the coupling of oxygen vacancies and Mn^2+^ to form *P_d_* and orienting along the *P_s_*. After the electric field is eliminated, *P_d_* can act as an internal field to restore the domain to its original state [88].

The volume effect of the defect dipole generates a restoring force through the alignment of the dipoles that causes the *P*_s_ to revert their initial direction, increasing the strain value [89]. Therefore, the defect dipole behaviors in various phase structures (formation, rolling-over, and migration diffusion) are to be studied, focusing on the intricate structural modifications of the BNT materials. It is very important to optimize strain performance by designing defects and utilizing the volume effect.

### 3.3. Phase Structure and Regulation of Defect Dipoles

The change in phase structure will also affect the strain. In Mn-BNT, Aksel et al. found that the sintering temperature would affect the valence state of Mn ions instead of the formation of any defect dipoles [90]. Zhang et al. revealed that the $Mn_{Ti}^{\prime \prime }$-$V_{O}^{••}$ defect dipole appeared in Mn-BNT-BT after annealing the material in a vacuum for a long time. They believe that the $Mn_{Ti}^{\prime \prime }$-$V_{O}^{••}$ defect dipole introduces a tetragonal structure field to increase the tetragonality of the material, thereby increasing the strain. While annealing in oxygen, Mn ions become Mn^4+^. The change in ion size regulates the relaxation degree of the material and increases the strain [91].

Jo et al. prepared Nb- and Fe-substituted BNT-BT-KNN ceramics and found that ${\left(Fe_{Ti}^{\prime }-V_{O}^{••}\right)}^{•}$ caused by acceptor doping would enhance the stability of the ferroelectric phase. They further studied Cu-BNT-BT-KNN and found that the existence of the $Cu_{Ti}^{\prime \prime }-V_{O}^{••}$ defect dipole also increased the stability of the ferroelectric phase [92,93]. However, the increase in the stability of the ferroelectric phase increases the *S*/*E* in Fe-substituted BNT-based materials and decreases the *S/E* in Cu-substituted materials. Li et al. introduced A-site vacancies into BNT-BKT materials and obtained a large strain with moderate hysteresis (*S* = 0.72%). It is considered that the $V_{A}^{\prime \prime }$-$V_{O}^{••}$ defect dipole introduces a random localized polarization field, which plays a nuclear role in the electro-induced phase transition and reduces the phase transition of the free energy [19]. In non-stoichiometric ceramics Bi_0.5+*x*_(Na_0.82_K_0.18_)_0.5−3*x*_TiO_3_ and Bi_0.5+*y*_(Na_0.82_K_0.18_)_0.5_TiO_3_, it is found that the generation of A-site vacancies will lead to the destruction of long-range ordered phases by random defect fields and make the domain switch easier [94]. Liu et al. studied nonstoichiometric 0.99Bi_0.505_(Na_0.8_K_0.2_)_0.5−*x*_TiO_3_-0.01SrTiO_3_ (BNKST (0.5−*x*)) ceramics and confirmed that the recoverable large strain caused by the reversible RE-FE phase transition is closely related to complex vacancy defects and local random fields [95].

Yao et al. found that in Mn-replaced A-site ions of BNT thin film, the presence of defect dipoles increased the size of PNRs. At the same time, the regulation of tolerance factors promoted the occurrence of oxygen octahedrons in-phase tilt. The phase structure of BNT itself is very sensitive to defects, and the phase structure is one of the determinants of strain characteristics. In addition to the general volume effect, domain effect, and grain boundary effect, the study of the mechanism of defect dipole behaviors in BNT materials should also consider the change of phase structure caused by the introduction of defect dipoles, including the change of average structure and local structure [96].

### 3.4. Defects in Conductive Characteristics

According to Hiruma et al., the volatilization of Bi ions in BNT-based materials brings in the generation of $V_{Bi}^{\prime \prime \prime }$ during the sintering process and further produces $V_{O}^{••}$ to counteract the charge. The leakage current increases and the domain boundary is pinned, making the material hard to polarize [97]. This served as the foundation for the regulation of various Bi/Na stoichiometric ratios and the preparation of non-stoichiometric BNT. It was discovered that the addition of excessive Bi reduced the leakage current of the material [98]. Li et al. further contend that the primary cause of the high leakage conductivity and challenging polarization of BNT materials is the long-range migration of $V_{O}^{••}$ due to the volatilization of Bi ions in BNT, which brings $V_{O}^{••}$ into the material. They prepared Nb-BNT, a perovskite ferroelectric material that differs from others in that donor doping causes a rapid increase in leakage current. Nb substitution decreases the leakage current in the materials by preventing the development of $V_{O}^{••}$. It is vital to take into account the change in conductivity properties brought on by defects since the strain must be acquired under the influence of a strong external electric field [99,100]. The conductivity mechanism of BNT has been studied from the nonstoichiometric and process aspects [101,102], but the research on the conductivity characteristics of BNT is still in its infancy and needs to be combined with the defect chemistry in different atmospheres [103,104].

### 3.5. Defects in Strain Fatigue Characteristics

The fatigue behavior of materials is closely related to a number of variables. Domain structure, including domain wall pinning and domain morphology, phase transition, and internal bias field, significantly influences the fatigue behavior of ferroelectric materials [105]. PZT-based ceramics are not entirely suited to BNT-based materials on account of the dramatic fluctuation of the fatigue properties between the NR and ER phases in BNTs [106,107]. The polarization will drop by 47.4% in 10^4^ cycles in the NR phase region [108], while the antifatigue of the ER region is enhanced by the lack of domain boundary structure characteristics. However, it is noticeable that the decrease in residual polarization and the increase in strain as the electric field cycles increase come from the phase transition during the fatigue process.

Using in-situ TEM, Guo et al. observed that the fatigue accompanying the increase in electric field cycles is due to the fragmentation of the domain brought about by the pinning of the defects on the domain wall. The excessive mobility of $V_{O}^{••}$, which was the fundamental defect in the material, affects the fatigue characteristics. $V_{O}^{••}$ will pin the intermediate state of polarization reversal, resulting in a deterioration of performance [109]. In BNT-SrTiO_3_, it was found that the improvement in fatigue performance comes from the decline in defect content [110]. Ehmke et al. discovered that the improvement in the fatigue properties of Cu-substituted BNT-BT was attributed to the change in the symmetrical structure and the reduction in the defect number by Cu substitution [111]. Shi et al. also found that the existence of ${\left(Fe_{Ti}^{\prime }-V_{O}^{••}\right)}^{•}$ defect dipoles in Fe-substituted BNT-BT improves its fatigue properties by suppressing the high mobility of $V_{O}^{••}$ [112]. Excellent fatigue resistance was achieved in Nb-doped, textured BNKT ceramics. The *S-E* curve was almost unchanged, and the performance did not noticeably degrade after 10^5^ cycles. This is because the formation of defect dipoles reduces the migration of oxygen vacancies and causes a decrease in the easily suppressed ferroelectric tetragonal phase. Additionally, the rising temperature increases the volume of a single cell, which improves the polarizability of the defect dipole and temperature stability [20].

These studies have demonstrated that the primary factor influencing the strain fatigue properties of the FE phase in BNT-based materials is the domain effect of material defects. There is a lack of macrodomain structure in the ER phase; thus, there is still a poor understanding of fatigue in this phase region.

## 4. Asymmetry Invoked by Defect Dipoles

**Figure 10:**
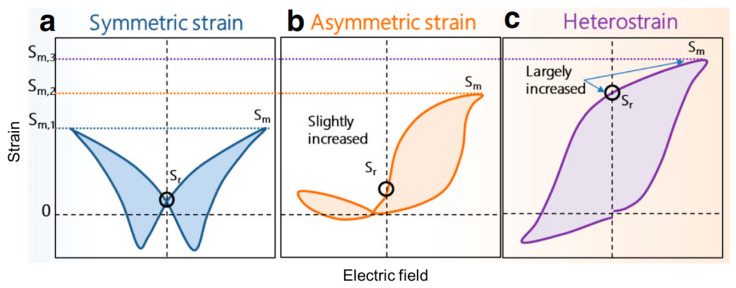
Three types of *S-E* curves: (**a**) symmetric strain; (**b**) asymmetric strain; and (**c**) heterostrain. Reproduced with permission from [49].

As mentioned above, vacancies will be frequently created in the perovskite structure of oxides. Through the regulation of defect dipoles, different types of strain curves can be achieved. Based on the latest research, the strain patterns include symmetric strain, asymmetric strain, and heterostrain, which are summarized in Figure 10a–c [12,113,114]. Most materials show a typical butterfly strain curve with a symmetric loop called symmetric strain. The residual strain (*S_r_*) is lower, and the maximum value (*S_m_*) is also lower. The properties of an asymmetric strain are that one side of the strain increases while the other side decreases, accompanied by a slight enhancement in both *S_r_* and *S_m_*. Heterostrain is a type of strain that is between symmetric and asymmetric. As the strain increases in the positive electric field direction and decreases in the negative electric field direction, it has a large *S_m_* and a significantly increased *S_r_*.

Giant asymmetric strain behaviors occur frequently in recently reported piezoceramics [115]. Liu et al. obtained a very large asymmetric strain (*S*/*E* > 1000 pm/V) in Nb-substituted [10] and Ta-substituted [21] BNT-based materials. This asymmetric strain is remarkably stable in the sample after polarization and aging. They suggest that there are some oriented ferroelectric domains in the material in which the defect dipoles formed by A-site vacancies play a pinning role. The strain asymmetry effect caused by the defect dipole was also observed in the A-site La ion-substituted BNT-BT [13] and Li-doped SBNT [114]. It is considered to be induced by a strong internal bias field (*E*_i_) formed by charged defects [116].

**Figure 11:**
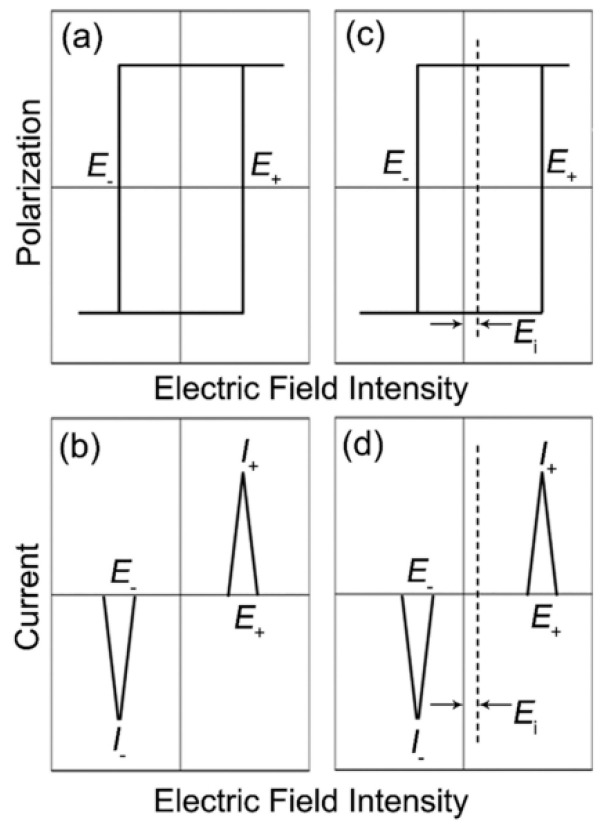
The asymmetry mechanism of the *P-E* curve and *I-E* curves. Reproduced with permission from [89].

In the hysteresis loop and current curves, the symmetric curves displayed the same coercive field (*E_c_*) under positive and negative electric fields (*E_+_* = *E_−_*), as shown in Figure 11a,b. Whereas, the *P-E* and *I-E* loops would show an asymmetric phenomenon after the poling process, accompanied by different *E_c_* (*E_+_* ≠ *E_−_*), as seen in Figure 11c,d. This asymmetric situation can be interpreted as meaning that as the *P_d_* is oriented along or close to the *P_s_* direction throughout the aging process, the *P_d_* will generate *E_i_* parallel to the polarization direction [117,118]. When an electric field is applied in the opposite direction of *E_i_*, the electric field should overcome the joint action of *E_c_* and *E_i_* to induce genesis domain switching, which will produce more *E_+_*. When the applied electric field is in the direction of *E_i_*_,_ the domain switch can be easily performed due to the contribution of *E_i_*, which has a smaller *E_−_*. The hysteresis loop and current curve shift as a result of *E_i_*. These conclusions apply to the strain curve as well, namely that the defect dipole induces the formation of the internal bias field, which leads to the shift of the *S-E* curve and the asymmetric response [119,120].

**Figure 12:**
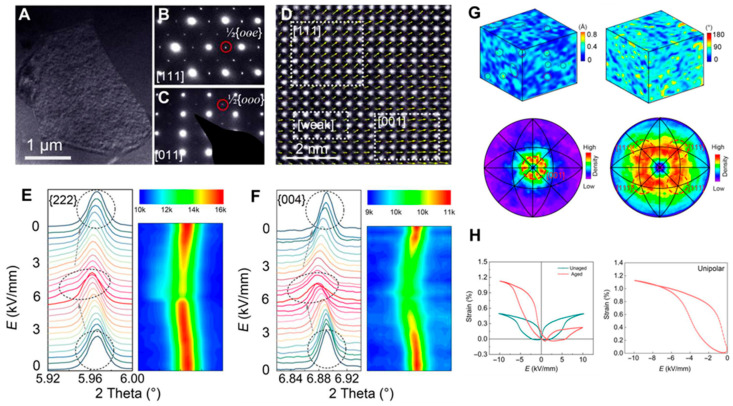
(**A**) TEM bright-field image of the global domain structure. (**B**,**C**) The corresponding SAED patterns of the nanodomains. (**D**) Polarization vector. (**E**,**F**) In-situ SXRD experiments. (**G**) 3D plots of the polarization magnitude and polarization vector for defect and electric dipoles. (**H**) *S-E* curves. Reproduced with permission from [121].

Recently, BaAlO_2.5_ with nominal oxygen defects was doped into BNT to produce a strongly polarized directional defect dipole, and a giant asymmetric strain was achieved [121]. The giant asymmetric strain is attributed to the reversible phase transition and the promotion of polarization rotation by the directional alignment of defect dipoles. That is, the introduction of BaAlO_2.5_ forms a strong polarized directional defect dipole, which can destroy the long-range order of ferroelectrics, resulting in ergodic relaxation behavior. This relaxation state is metastable, and it is easy to transform into an ordered ferroelectric state when the electric field is loaded. The relaxation of the ferroelectric phase transition can cause a large strain. Meanwhile, the directional defect dipole forms an internal bias field, and the internal bias field will cause different degrees of polarization rotation in different electric field directions. When the electric field is the same as the initial polarization direction of the defect dipole, polarization rotation is more likely to occur, resulting in asymmetric strain. The coexistence of *P*4*bm* and *R*3*c* phases is confirmed by the nanodomains in TEM and the superlattice spots observed in the corresponding SAED patterns. Moreover, the overall disordered distribution induced by defect dipoles is observed by polarization vector mapping, as illustrated in Figure 12a–d. Meanwhile, in-situ synchrotron x-ray diffraction (SXRD) is carried out to imply a reversible phase transition from *P*4*bm* to *R*3*c*, as illustrated in Figure 12e,f. The polarization rotation is accelerated when the dipole is aligned along the applied electric field. The defect dipole has a much stronger polarization than the electric dipole, which may produce a substantial pinning effect, as pointed out in Figure 12g. The different polarization directions of the defect dipole and the electric dipole reveal that the long-range order is disrupted, which provides the internal structural basis for the large strain [122,123]. In addition, the introduction of defect dipoles forms an internal bias electric field, which leads to unequal lattice strain in different electric field directions, yielding an asymmetric strain of up to 1.12% (Figure 12h) [121].

**Figure 13:**
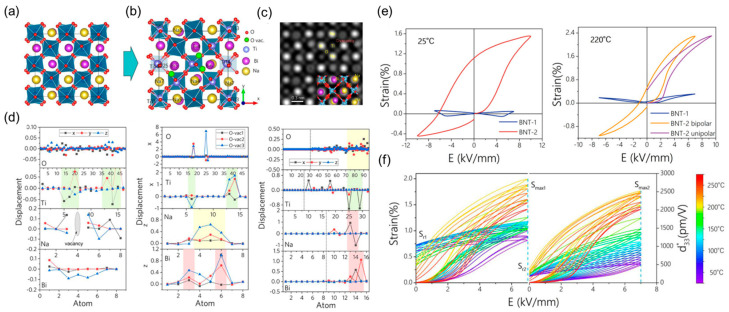
The crystal structure of BNT with (**a**) zero and (**b**) 3/48 oxygen vacancies. (**c**) Simulated HAADF STEM image of BNT containing 3/48 oxygen vacancies. (**d**) The relative relaxation ratio is induced by oxygen vacancies. (**e**) The strain curves of BNT at 25 °C and 220 °C. (**f**) The strain curves of two samples with increasing temperature. Reproduced with permission from [12].

In addition to the symmetric or asymmetric strain, through defect engineering and domain engineering design, Feng et al. achieved a heterogeneous strain between the symmetric and asymmetric strains [12]. The strong coupling of large ion displacement, oxygen octahedral rotation, and energy level shift is caused by vacancies and electric fields. These will improve the stability of disordered structures and generate non-uniform domains as exhibited in Figure 13a–d [124], triggering an ultra-high electrical strain of 2.3% at high temperature (220 °C), which significantly surpasses the state-of-the-art piezoceramics, as shown in Figure 13e,f.

**Figure 14:**
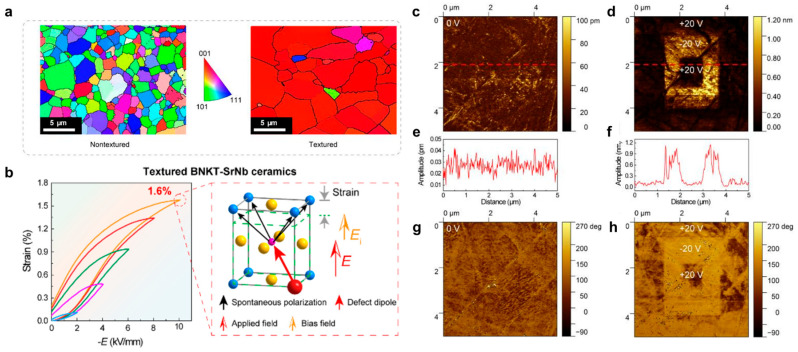
(**a**) EBSD of the textured and nontextured BNKT-SrNb ceramics. (**b**) Strain properties of the textured BNKT-SrNb ceramic. The evolution of domain structure under the different electric fields for BNKT-SrNb ceramics. PFM amplitude images under (**c**) 0 V and (**d**) 20 V. Piezoresponse amplitude profiles were generated from the red dotted line under (**e**) 0 V and (**f**) 20 V. Phase images under (**g**) 0 V and (**h**) 20 V. Reproduced with permission from [22].

Moreover, the reaction templated grain growth method (RTGG) was employed to prepare oriented Sr/Nb-doped Bi_0.5_(Na_0.82_K_0.18_)_0.5_TiO_3_ (BNKT-SrNb)-textured ceramics with good fatigue resistance and low hysteresis [22]. The electron backscatter diffraction confirmed that it has a high <00l> preferential orientation, which can tremendously benefit the polarization rotation under the electric field, as shown in Figure 14a. The oriented $V_{A}^{\prime \prime }-Nb_{Ti}^{•}$ defect dipoles were formed by introducing Nb^5+^ ions. A large internal bias electric field was generated due to the alignment of the defect dipole along the polarization direction, resulting in asymmetric strain and a huge unipolar strain (1.6%), as seen in Figure 14b. PFM was used to reveal the asymmetric strain behavior under the applied voltage of ±20 V, as shown in Figure 14c–h. The application of −20 V indicates the formation of a macroscopic ferroelectric phase, while the application of +20 V cannot induce a complete transition from the ergodic relaxation state to the ferroelectric phase. The evolution of domain structure confirmed the existence of an internally biased electric field at microscopic and macroscopic levels. It demonstrated that the synergistic contribution of reversible electric field-induced phase transition, grain orientation engineering, and defective dipole engineering is the reason for the significant enhancement of strain.

**Figure 15:**
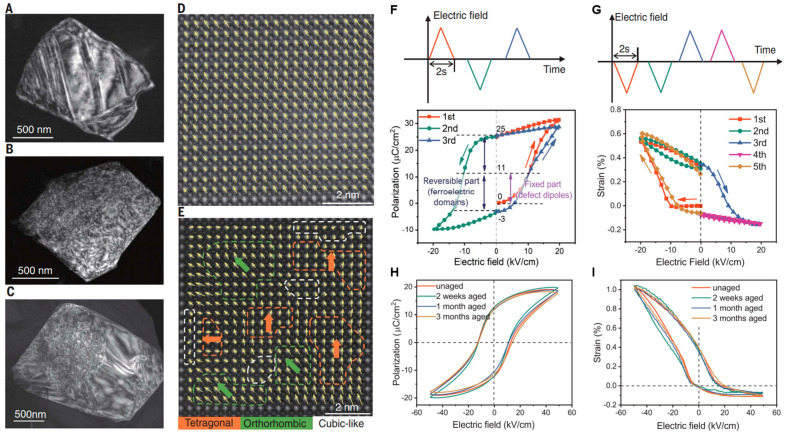
TEM observations of ferroelectric domain changes in (**A**) stripe, (**B**) nanosized, and (**C**) mixed domains. (**D**,**E**) HAADF images of a stripe domain and a nanosized domain region. (**F**,**G**) Unipolar measurement performed within 2 s, and *P−E* and *S−E* behaviors observed after multiple measurement. (**H**,**I**) *P−E* loops and *S−E* curves during 3 months aging process. Reproduced with permission from [125].

In addition to BNT-based materials, the asymmetric strain properties of other piezoelectric materials can also be affected by defect dipoles. For KNN-based ceramics, Guo et al. elucidated the existence of asymmetric strain behavior when defect dipoles are introduced [29,125]. TEM was employed to observe the switching of several ferroelectric domains under the electric field, as illustrated in Figure 15a–c. The nanodomain region suggests multiphase coexistence in nanosized particles that is beneficial to the rotation and arrangement of defect dipoles along the electric field, as shown in Figure 15d,e. So the defect dipoles can be aligned by the initially applied external electric field in this region, and significant lattice distortion is further induced. Simultaneously, the directional alignment of defect dipoles leads to significant fixed polarization (Figure 15f,g). The lattice distortion caused by the oriented defect dipole under the electric field and the distortion invoked by the ferroelectric domain reversal are coupled with each other, generating the asymmetric strain curve of K_0.485_Na_0.485_Sr_0.03_NbO_3_ (KNSN3) ceramics. Furthermore, the polarization and strain properties of KNSN3 remain stable during the aging process, as exhibited in Figure 15h,i, indicating that the defect dipole can have excellent stability after orientation under the initial electric field.

## 5. Summary and Prospects

In this paper, the effect of defect dipole behavior on the structure and strain evolution of BNT-based piezoceramics is reviewed, including the volume effect, domain effect, and grain boundary effect. Associated with the conductive and fatigue characteristics, it is of great significance to obtain BNT-based materials with optimized strain properties such as large *S*/*E*, small hysteresis, and fatigue resistance. This review provides a promising strategy for achieving giant strain, especially in the formation of asymmetry, by utilizing the defect dipole that is induced by ion substitution and process treatment. However, there are still the following issues in the defect regulation of the ceramics: (1) The determination of defect structure as well as the formation, distribution, flipping, and diffusion migration behavior of defect dipoles are still insufficient. (2) The structure and strain regulation mechanisms of defect dipoles in different phase regions are not clear. The mode of action of defects on the bulk effect of PNRs is unknown. (3) In the fatigue process of the ER phase with phase transition characteristics, the role of defects needs to be resolved. Through the defect design with different valence at the A/B sites, the defect dipole behavior, structure (phase structure, domain structure, and microstructure), and strain performance (strain magnitude, hysteresis, and fatigue characteristics) by means of atmosphere treatment and process optimization should be further explored so as to achieve comprehensively large strain with small hysteresis and antifatigue performance in BNT-based materials. Until now, the universal conclusion that there is a lack of in-depth details and quantification has always been drawn from the previous reports. The regulation mechanisms and their dynamics in defect dipoles are still to be fully understood in both local and average structures.

## Figures and Tables

**Table 1:** Electric field-induced strain properties of lead-free piezoceramics.

Compositions	*E*_max_ (kV/cm)	*S*_max_ (%)	d_33_* (pm/V)	Hysteresis	Symmetry	Methods
BNT-Nb [10]	50	0.7	1400	large	asymmetric	SR
BNT-Sr [11]	80	0.1	125	negligible	symmetric	SR
BNT-2 [12]	70	(220 °C) 2.3	3286	moderate	heterogeneous	SR
BNT-BT-SBT [9]	80	0.152	190	negligible	symmetric	SR
BNT-BT-La [13]	75	0.53	707	moderate	asymmetric	SR
BNT-BT-Zr [14]	40	0.23	573	large	symmetric	SR
BNT-BT-LN [15]	50	0.64	1280	small	symmetric	SPS
BNT-BT-SS [16]	85	1.89	2230	large	asymmetric	SR
BNT-BT-FN [17]	40	0.36	907	large	symmetric	TGG
BNBT-YT [18]	52	0.37	708	large	asymmetric	SR
BNKT-SBTZ [19]	110	0.72	655	moderate	symmetric	SR
BNKT-Nb [20]	60	0.65	1083	moderate	asymmetric	RTGG
BNKT-Ta [21]	50	0.62	1240	large	asymmetric	SR
BNKT-SrNb [22]	100	1.6	1600	moderate	asymmetric	RTGG
BNT-BKT-BT [23]	40	(90 °C) 0.41	1030	large	symmetric	RTGG
BNT-BKT-BA [24]	40	0.398	995	large	symmetric	RTGG
BNT-ST-NN [25]	30	0.25	833	large	symmetric	SR
BNT-ST-FN [26]	70	0.26	371	small	symmetric	SR
BNT-ST-MnO_2_ [27]	20	0.168	840	large	symmetric	SR
BNT-BST-BT-ZS [28]	120	0.24	200	negligible	asymmetric	SR
KNSN/NiO [29]	50	1.35	2700	moderate	heterogeneous	TGG
KNN-Cu [30]	50	0.5	1000	large	asymmetric	SR
KNNS-BNZ [31]	30	0.14	493	large	symmetric	SR
BT-Mn [32]	50	0.23	460	moderate	symmetric	SR
BT-LH [33]	60	0.2	334	moderate	symmetric	SR
BF-BT-Fe [34]	50	0.34	680	large	symmetric	SR

## Data Availability

Not applicable.
